# Real-time reorientation and cognitive load adjustment allow for broad application of virtual reality in a pediatric hospital

**Published:** 2021-11-06

**Authors:** Thomas J. Caruso, Ahtziri Fonseca, Ariana Barreau, Michael Khoury, Maria Menendez, Ellen Wang, Kiley Lawrence, Christian Jackson, Samuel Rodriguez

**Affiliations:** ^1^Department of Anesthesiology, Perioperative, and Pain Medicine, Stanford University School of Medicine, Stanford, CA, 94304, USA; ^2^Stanford Chariot Program, Lucile Packard Children’s Hospital, Palo Alto, CA, 94305, USA; ^3^Department of Anesthesiology, Perioperative, and Pain Medicine. Massachusetts General Hospital. Boston, MA, 02114, USA

**Keywords:** virtual reality, therapeutics, pediatrics

## Abstract

**Background:**

With a new generation of affordable portable virtual reality (VR), clinicians are discovering more utility for VR, while also identifying opportunities for improvement, such as the inability to reorient the horizon line during repositioning or transport, or modulate cognitive load in real time.

**Aim:**

At our institution, this lack of functionality prohibited or decreased VR usage in some clinical scenarios such as dressing changes with dynamic positioning. The purpose of this brief report is to describe the development and use of a VR application that is optimized for the healthcare setting and report historical effects of patients who utilized VR as supplement to Child Life procedures. Eligible affects per chart review included Happy, Relaxed, Anxious, Distressed, Unable to Assess.

**Materials and Methods:**

Given the need for real-time reorientation and cognitive load modulation, we created the Space Pups™ VR application. The experience was launched as part of the Stanford Chariot Program in the summer of 2017, and its usage was tracked through the electronic medical record and a VR application dashboard. Chart review was queried from 3 January 2018 to 9 August 2021 for pediatric patients who used VR with real-time reorientation and cognitive load modulation as a supplement to their Child Life interventions.

**Results:**

The Space Pups™ experience has been successfully used in a variety of settings, including perioperative care, vascular access, wound care, and ENT clinic, a total of 1696 times. Patients ranged from 6 years to 18-year old, with no reports of side effects. Significant results (*P*<0.001) were observed pre- and post-VR use for affect improvements in Happy, Relaxed, and Anxious, but not for Distressed.

**Conclusions:**

The ability to reorient VR experiences in real time has increased functionality where other applications have failed.

**Relevance for Patients:**

While more studies are needed to quantify the anxiolytic and pain-reducing effect of Space Pups™, our report demonstrates the feasibility of this VR experience as a non-pharmacological modality to safely increase patient cooperation in a wide variety of clinical settings.

## 1. Introduction

Pediatric patients with untreated pain and anxiety during medical procedures may experience short- and long-term consequences, including post-traumatic stress disorder, needle phobia, and lack of trust of healthcare providers [1]. Virtual reality (VR) has emerged as a promising non-pharmacologic tool for reducing pain and anxiety in some adults and children [2,3]. Children, in particular, can benefit greatly from VR as a distractive tool to minimize attention to aversive stimuli by focusing on a 3-dimensional interaction of a computer-generated environment. Recent studies have citied large effect sizes in overall pain and anxiety reduction with VR use in pediatric patients undergoing a wide range of medical procedures such as venous access, burn, and oncological care [4,5]. VR has also been reported as an effective anxiolytic adjunct in a variety of settings, including phlebotomy, wound care, and chronic pain rehabilitation with additional potential to reduce the need for opioid-based analgesia [2,3,5,6]. Given the availability of low cost, portable, head-mounted VR units, clinicians have novel opportunities to integrate VR therapy into a wide range of clinical modalities. However, given the paucity of studies focusing on the clinical relevance of VR therapy within pediatric populations, it is imperative to further elucidate and address any shortcomings in a clinical context [7].

Most VR experiences are designed for users to play in a fixed viewing direction, usually seated or standing. This feature limits its application in healthcare settings given that many patients are supine. Furthermore, many patients experience fluctuations of noxious stimuli during encounters that include dressing changes, minor procedures, and phlebotomy. A VR application that provides healthcare workers the ability to modulate gameplay as stimuli change allows for increased distractibility at opportune moments. We introduce a novel VR experience that can reorient the horizon line in real time to allow for gameplay in any position with cognitive load modulation.

## 2. Materials and Methods

### 2.1. Development of VR application

The Space Pups™ VR experience was developed by a team of physicians, research fellows, and a software engineer through the Chariot Program at Lucile Packard Children’s Hospital Stanford. Initial design considerations were developed in consultation with patients, parents, and pediatric psychologists. The process included five iterative revisions based on their feedback. During each round of feedback, adjustments were made to the software to (1) increase cognitive load demand during provider-initiated accelerated gameplay; (2) improve the gaming motivation to progress through levels; (3) optimize the audio accompaniment to the application; and (4) insert additional rewards for users based on game progression. Optimization of gameplay was completed when consensus was reached between the developers and providers that the cost of further iterations did not dramatically increase perceived patient benefit.

**Figure 1:**
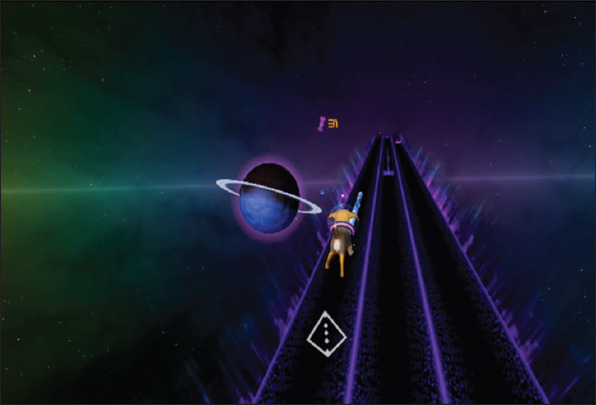
With subtle head movements (looking left or right), the player directs the character to the treats, which are laid out to the beat of the music.

**Figure 2:**
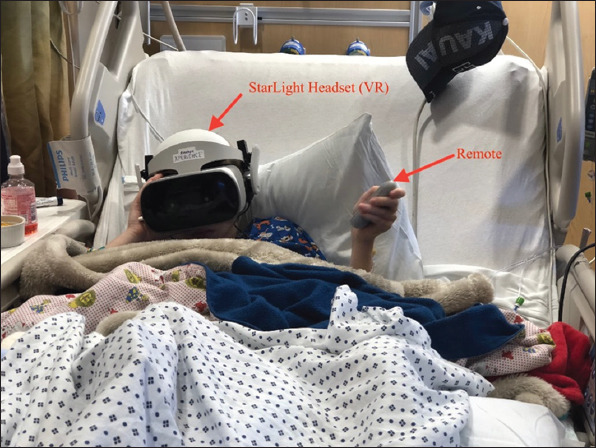
Patient playing Space Pups™ as the nurse prepares to give medication. Once the character is chosen, the patient uses either head movement or the remote to navigate his character in Space Pups™.

During the Space Pups™ experience, patients choose from one of five different pups that they steer down a highway in outer space, collecting treats from one of three highway lanes that synchronize with the beat of music (Figure 1). The patient or clinician can change the horizon line orientation of the highway at any point during the game by triple swiping the side finger pad on a Samsung Gear VR or by holding down the App button (center button) on the Lenovo Mirage remote controller (Figure 2). Patients were excluded from VR use if they had any of the following- significant cognitive impairment, history of severe motion sickness, current nausea, prone to seizures, visual problems, clinically unstable, or required urgent/emergent intervention.

The experience is played either by gaze (lane changes are accomplished by rotating their head 5° left or right) or controller for patients with limited head mobility or whose heads are required to be still for clinical procedures, such as nasal endoscopy. Intuitive gameplay and minimization of menus allow the patient to be fully immersed in the game during minor procedures. The scoring system (point accumulation based on the number of treats retrieved) encourages patients to remain focused on the game to reach a high score. There is no fail state, allowing users to remain in the game continuously. In addition, a mechanism to transiently increase cognitive load for 12 s initiates a visual vortex and increases the frequency targets during times of increased pain or stress [6]. This is important to the game’s mechanics because it allows for further distraction and cognitive load modulation during periods of potentially increased pain or anxiety.

### 2.2. Case

An 11-year-old boy was admitted to the patient care unit with a broken right leg, requiring multiple procedures and dressing changes. Written informed consent was obtained from the patient’s guardians, as well as an assent from the patient. Space Pups™ was loaded in the VR headset. Pain and anxiety scores were monitored through self-reported questionnaires before and after VR use. The VR headset and game were initially presented to the patient as a source of entertainment to increase comfort during his recovery. After the patient became comfortable with Space Pups™, it was used to alleviate pain and anxiety during wound dressing changes. The horizon reorientation allowed the patient to continuously play while he was being moved from supine and fowler position during his dressing changes. During periods of potentially increased pain and anxiety, the providers would initiate the visual vortex within the gameplay to increase cognitive load and further immerse the patient. The VR headset was used daily by the patient for 4 weeks during his recovery.

### 2.3. Effectiveness

Through partnerships with Child Life Specialists, nurse specialists, and physicians, the Chariot Program provided a combination of Gear VR and Lenovo headsets to multiple healthcare environments within Lucile Packard Children’s Hospital Stanford. Through electronic medical record (EMR) integration with the Child Life note and the headset interface alongside self-reported questionnaires, side effects and usage were analyzed.

After obtaining IRB approval, the EMR was queried from 3 January 2018 to 9 August 2021 for patients who used VR as a supplement to their Child Life interventions. Patient affect was measured pre- and post- VR utilization by Child Life Specialists. Chi-square tests for equivalent proportions (*P*<0.0001) were performed to determine affect count differences between both groups (pre- vs post-VR).

## 3. Results

Before using the Lenovo VR headset, the patient had never experienced VR. Despite its novelty, he learned how to choose a character and play the game within minutes, with limited instruction. By intentionally developing the game with limited head movement, nurses were able to successfully change his dressings while he remained in game play. The patient explained that pain and anxiety during dressing changes were significantly lower when using the headset. The patient’s parents stated that they would recommend VR for other anxious children and expressed how thankful they were that their son was able to use the VR headset.

**Figure 3:**
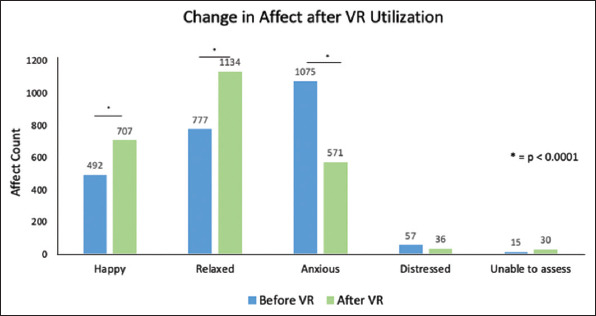
Pre- vs Post-VR affect count for 1696 patients. Chi-square test for equivalent proportions (*p*<0.0001) revealed significant differences between Happy vs Not Happy, Relaxed vs Not Relaxed and Anxious vs Not Anxious responses with no significant differences between Distressed vs Not Distressed and Unable to Assess vs Able to Assess.

Space Pups™ has been used in eight different clinical settings a total of 1696 times with an average in-application duration of 4.56 min (Table 1). There have been no reports of nausea, motion sickness, or dizziness on review of Child Life notes and self-reported questionnaires. Significant differences (*P*<0.0001) in affect pre- versus post- VR use were observed between Happy versus Not Happy, Relaxed versus Not Relaxed, and Anxious versus Not Anxious responses with no significant differences between Distressed versus Not Distressed and Unable to Assess versus Able to Assess (Figure 3).

**Table 1:** Clinical contexts for VR reorientation

Vascular Access/Phlebotomy
Dental Procedures
Perioperative setting
Nasal Endoscopy
Vascular Access
Epidural Placement
Dressing Changes
Wound Care

## 4. Discussion

This report highlights the successful application of VR in the clinical setting, utilizing horizon line reorientation and cognitive load modulation. By increasing cognitive load and reducing attention to aversive stimuli, the patient reported reduction of subjective pain intensity, and in-application reorientation allowed for uninterrupted care. The application has been widely and successfully used in a variety of settings.

Although VR and other immersive technologies have previously been studied as non-pharmacologic adjuncts for the modulation of pain and anxiety in clinical contexts, the inherent limitations of many VR games – specifically, the inability to reorient the horizon for patients in non-standard gaming positions – limit the patient population that can safely and easily utilize this technology. Cognitive load modulation has previously been shown to subjectively reduce one patient’s anxiety during a vascular access procedure [8], but its use has not yet been extended into broader clinical contexts. To the best of our knowledge, this is the first report showing the broad clinical applicability of horizon line reorientation and cognitive load modulation in VR with chart review usage to support the clinical efficacy of VR as a means to increase positive affect in various clinical settings.

Future studies will quantify the anxiolytic and analgesic effect of Space Pups™. This case study demonstrates the use of this VR game as a non-pharmacological treatment to distract patients during wound care. The game can accommodate different clinical contexts due to horizon line reorientation and provides increased distraction during increased cognitive loads.
